# Analog Forecasting of Extreme‐Causing Weather Patterns Using Deep Learning

**DOI:** 10.1029/2019MS001958

**Published:** 2020-02-23

**Authors:** Ashesh Chattopadhyay, Ebrahim Nabizadeh, Pedram Hassanzadeh

**Affiliations:** ^1^ Department of Mechanical Engineering Rice University Houston TX USA; ^2^ Department of Earth, Environmental and Planetary Sciences Rice University Houston TX USA

**Keywords:** extreme weather events, deep learning, analog forecasting, weather prediction, data‐driven modeling

## Abstract

Numerical weather prediction models require ever‐growing computing time and resources but, still, have sometimes difficulties with predicting weather extremes. We introduce a data‐driven framework that is based on analog forecasting (prediction using past similar patterns) and employs a novel deep learning pattern‐recognition technique (capsule neural networks, CapsNets) and an impact‐based autolabeling strategy. Using data from a large‐ensemble fully coupled Earth system model, CapsNets are trained on midtropospheric large‐scale circulation patterns (Z500) labeled 0–4 depending on the existence and geographical region of surface temperature extremes over North America several days ahead. The trained networks predict the occurrence/region of cold or heat waves, only using Z500, with accuracies (recalls) of 69–45% (77–48%) or 62–41% (73–47%) 1–5 days ahead. Using both surface temperature and Z500, accuracies (recalls) with CapsNets increase to ∼80% (88%). In both cases, CapsNets outperform simpler techniques such as convolutional neural networks and logistic regression, and their accuracy is least affected as the size of the training set is reduced. The results show the promises of multivariate data‐driven frameworks for accurate and fast extreme weather predictions, which can potentially augment numerical weather prediction efforts in providing early warnings.

## Key Points

- A data‐driven extreme weather prediction framework based on analog forecasting and deep learning pattern‐recognition methods is proposed
- Extreme surface temperature events over North America are skillfully predicted using only midtropospheric large‐scale circulation patterns
- More advanced deep learning methods are found to yield better forecasts, encouraging novel methods tailored for climate/weather data

## 1 Introduction

Predicting extreme weather events such as heat waves and cold spells is of significant scientific and societal importance. However, despite decades of progress in weather prediction, mostly through improving computationally demanding numerical weather prediction (NWP) models and data assimilation techniques (Alley et al., 2019; Bauer et al., 2015), forecasting the anomalous atmospheric circulation patterns that often drive these extreme events has remained a challenge. For example, blocking events, which are large‐scale, persistent, high‐pressure systems that block/divert the usual eastward winds (Woollings et al., 2018), have caused some of the most devastating natural disasters in recent times such as the 2003 and 2010 heat waves in Europe (Barriopedro et al., 2011; Woollings et al., 2018). Yet the state‐of‐the‐art NWP models have difficulties with accurately predicting the formation and persistence of blocking events (Ferranti et al., 2015; Matsueda, 2011; Pelly & Hoskins, 2003). Overall, the key characteristics of extreme‐causing weather patterns, their dynamics, and conditions that favor their formation (i.e., precursors) are not well understood (Coumou et al., 2015; Hassanzadeh et al., 2014; Horton et al., 2015; Hassanzadeh & Kuang, 2015; McKinnon et al., 2016; Nakamura & Huang, 2018; Nabizadeh et al., 2019; Teng et al., 2013; Woollings et al., 2018).

Recent advances in artificial intelligence have revolutionized how problems in various domains of business and science are approached (Goodfellow et al., 2016; LeCun et al., 2015). For example, in climate science, using machine learning techniques to accurately and efficiently represent unresolved physical processes in the atmosphere and ocean has produced promising results (Brenowitz & Bretherton, 2018; Bolton & Zanna, 2019; O'Gorman & Dwyer, 2018; Rasp et al., 2018; Salehipour & Peltier, 2019) and has the potential to significantly improve climate modeling and long‐term climate projections in the coming years (Chattopadhyay et al., 2019; Gentine et al., 2018; Reichstein et al., 2019; Schneider et al., 2017). Moreover, deep learning techniques have been very successful in predicting some types of sequential data (Goodfellow et al., 2016). Consequently, whether such techniques can be used for data‐driven forecasting of the spatiotemporal evolution of the weather systems (and their extreme events), for example, after training on high‐resolution NWP model outputs or observational data, has become an active area of research. Recent efforts pursuing this approach, which essentially requires a neural network to accurately, for some time, emulate the high‐dimensional nonlinear dynamics governing the evolution of the turbulent atmospheric circulation, have shown the promises and challenges of this approach (Chattopadhyay et al., 2019; Dueben & Bauer, 2018; Scher, 2018; Scher & Messori, 2019; Vlachas et al., 2018; Weyn et al., 2019).

In the current study, for data‐driven prediction of extreme‐causing weather patterns, we introduce an alternative framework that is based on analog forecasting, that is, making prediction by finding similar pattern(s), or analog(s), in the past (Lorenz, 1969; Van den Dool, 2007). In the historical context, before the advent of powerful electronic computers and stable numerical schemes for integrating the partial differential equations of the NWP models, analog forecasting was a key tool in weather prediction; for example, it was used in the planning of the D‐Day for the 1944 Normandy invasion (McDermott & Wikle, 2016). Analog forecasting was used less frequently in later decades, due to the challenges in finding useful analogs and the rapid growth of NWP (Van den Dool, 2007), although the approach has the potential for a comeback given the rapid increase in data and emergence of new auxiliary methods (McDermott & Wikle, 2016; Zhao & Giannakis, 2016).

Here, we build our data‐driven framework on analog forecasting because the patterns of the circulation, for example, the relative positions of high‐ and low‐pressure systems, play a key role in the spatiotemporal evolution of the circulation and the initiation of extreme events at the surface, and analog forecasting essentially casts weather prediction as a complex pattern‐recognition problem, an area that has been truly revolutionized by deep learning in recent years (Goodfellow et al., 2016; LeCun et al., 2015). Rather than looking for one perfect analog or a combination of near‐perfect analogs that are identified, for example, based on pattern correlation or Euclidean distance in the grid space, as pursued in traditional analog forecasting (Van den Dool, 1994, 2007), our framework employs deep learning techniques to classify the patterns based on their key, likely a low‐dimensional, set of features and decipher the complex relationship between this feature space (at the altitude of ∼5 km) and the extreme events (at the surface) among all training samples. The purpose of this paper is to provide a proof of concept for this framework.

The structure of the paper is as follows. In Section 2.1, the data and definitions of extreme events and their onsets are presented. In Section 3, the data‐driven extreme weather prediction framework, including the labeling, training, and testing procedures, are discussed. Results are presented in Section 4 followed by a discussion in Section 5.

## 2 Data

### 2.1 LENS Data

We use daily data from the large‐ensemble (LENS) Community Project (Kay et al., 2015), which consists of a 40‐member ensemble of fully coupled atmosphere‐ocean‐land‐ice Community Earth System Model Version 1 (CESM1) simulations with 1920–2005 historical radiative forcing. For each ensemble member, the same historical radiative forcing is used, but random, weak perturbations are added to the initial state of each member to create an ensemble. To ensure abundant training samples for the purpose of demonstrating a proof of concept for the framework, we choose to use data from a LENS climate model, rather than reanalysis data (see Section 5). Still, the simulated atmospheric circulation is nonstationary, turbulent, and multiscale, with complexities similar to those of the real atmosphere, thus providing a challenging testbed for our data‐driven extreme weather prediction framework.

From this data set, we use surface air temperature, measured as temperature at 2 m above ground (T2m), and geopotential height at 500 mb (Z500). We use the daily averaged T2m and Z500 from 1920–2005 for the months of June–August (boreal summer) and December–February (boreal winter) from all 40 ensemble members.

### 2.2 Extreme Hot and Cold Events and Their Onsets

We focus on extreme temperature events over the North American continent in the subtropical and midlatitude regions between 30°N and 60°N. For a given calendar date in a given ensemble member, the T2m anomalies are computed by removing the climatological mean, defined as the 15‐day running mean of T2m centered around that date and averaged over all ensemble members. Following Chan et al. (2019), heat waves (cold spells) are defined as land grid points over North America in summer (winter) with daily T2m anomaly in the 99th (1st) percentile and larger (smaller) than 3 K ( −1 K) for a sequence of at least five consecutive days. We then identify the onsets of these extreme temperature events as the first day of each sequence.

Unlike data commonly used in deep learning applications, climate data have spatiotemporal correlations, which can affect the training process (e.g., by reducing the effective sample size) and/or can lead to artificially high accuracies during testing (e.g., if the training and testing sets have strongly correlated samples). Here we aim to choose distinct samples of onsets with minimal correlations within and across the training and testing sets. In constructing the data set onsets to be used for training/testing, we follow these criteria: We include an onset if there is no other extreme events within the earlier 5 days and if it is not followed by another extreme event within the next 10 days (i.e., maximum of one onset in each 16‐day window over North America). This procedure substantially removes potentially (temporally) correlated Z500 patterns corresponding to persistent heat waves/cold spells that may artificially enhance prediction skills. We have experimented with the size of this window ranging from 16–24 and found no noticeable changes in the reported accuracies. For non‐extreme events, we ensure that their T2m anomaly is not in the 99th (1st) percentile and that they are not chosen from the events in the 16‐day windows defined above.

As discussed later, we randomly divide the samples in this data set of onsets (generated using all 40 ensemble members) to a training set and a testing set. To absolutely ensure that the reported accuracies are not contaminated by any temporal correlation between the samples in the training and testing sets (even after the procedure described above), we conduct another analysis in which data from 30 ensemble members are used in the training set and data from the 10 remaining ensemble members are used in the testing set (thus absolutely no temporal correlation between the training and testing samples). The analysis demonstrates the same accuracies as those reported in the paper.

### 2.3 Geographical Clustering of the Extreme Events' Onsets

**Figure 1:**
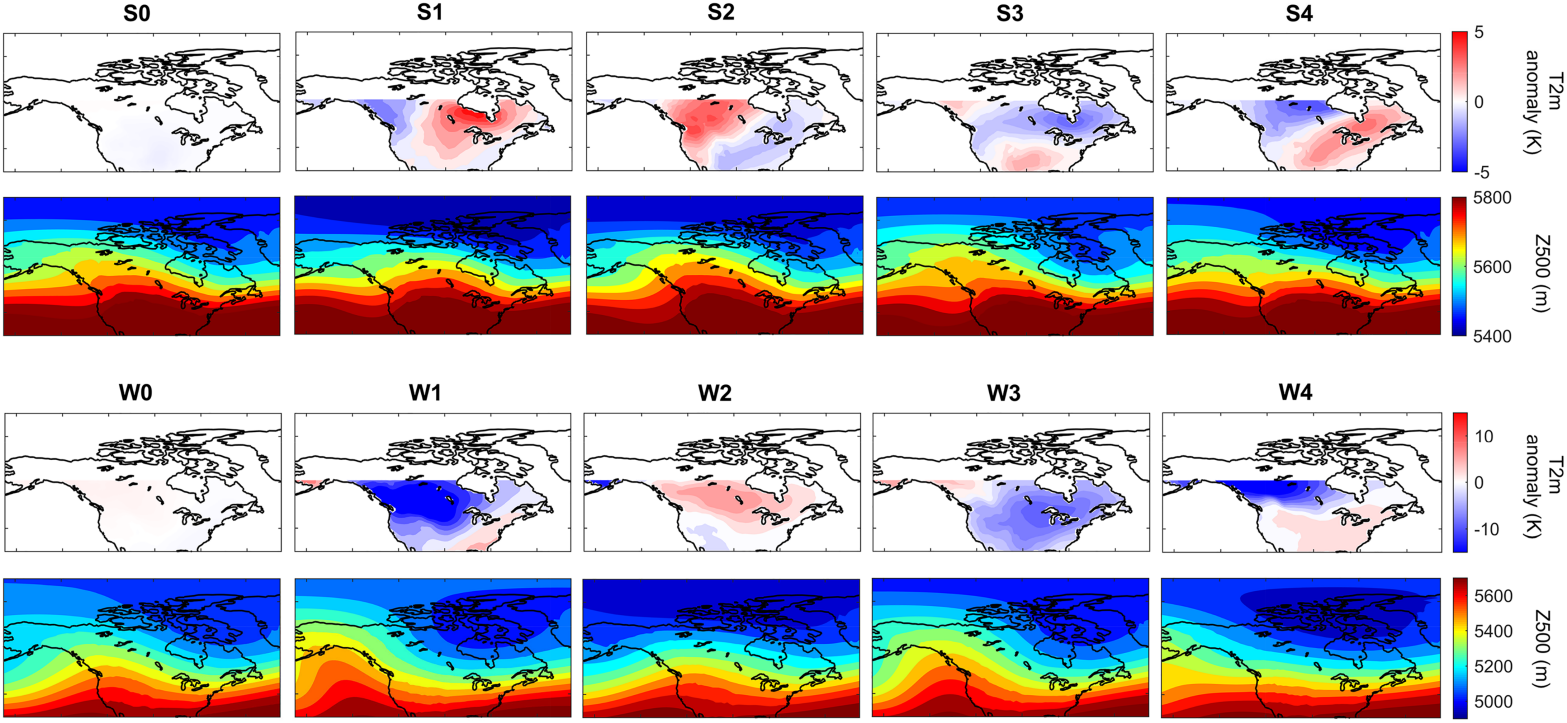
Cluster centers of T2m anomalies at the onsets and Z500 patterns of 3 days earlier. The top (bottom) two rows correspond to summers (winters). S0 (W0) shows the average of T2m and Z500 patterns from days with no heat wave (cold spell). S1–S4 and W1–W4 are obtained from K‐means clustering the anomalous T2m patterns at onsets into four classes, which roughly separates the extreme events into four geographical regions: Northern Canada (S1), Western United States‐Canada (S2), Southern United States (S3), and Eastern United States‐Canada (S4) in summers and Northwest United States‐Canada (W1), Alaska (W2), Northeast United States‐Canada (W3), and Northern Canada (W4) in winters. Rows 1 and 3 show the cluster centers, while rows 2 and 4 show the average of Z500 patterns 3 days before the onsets for each cluster.

We cluster the onsets of extreme events into four distinct yet cohesive geographical regions, separately for winters and for summers. Following Vigaud et al. (2018), an empirical orthogonal function analysis is first performed on the T2m patterns on the onset days and the first 22 principal components, which explain over 90% of the variance, are retained. The K‐means algorithm (Lloyd, 1982) is then used on the retained principal components and repeated 1,000 times with new initial cluster centroid positions, and a Cluster Index 1, 2, 3, or 4 is assigned to each day. Rows 1 and 3 of Figure 1 show the cluster centers (in terms of T2m).

The aim of our data‐driven framework is to predict whether in a few days, a Z500 pattern would lead to no extreme event (assigned Cluster 0) or an extreme event in the geographical regions corresponding to Clusters 1–4. Note that here we focused on four clusters to have a balance between too many clusters that may not be distinct or too few that would not effectively separate the geographical regions and demonstrate the effectiveness of the proposed data‐driven extreme weather event prediction framework. Also, here we use K‐means, a simple unsupervised algorithm, but other methods such as hierarchical clustering or self‐organizing maps (Cheng & Wallace, 1993; Mo & Ghil, 1988; Horton et al., 2015) could be used instead.

## 3 Methodology

### 3.1 Deep Learning Techniques: ConvNet and CapsNet

**Figure 2:**
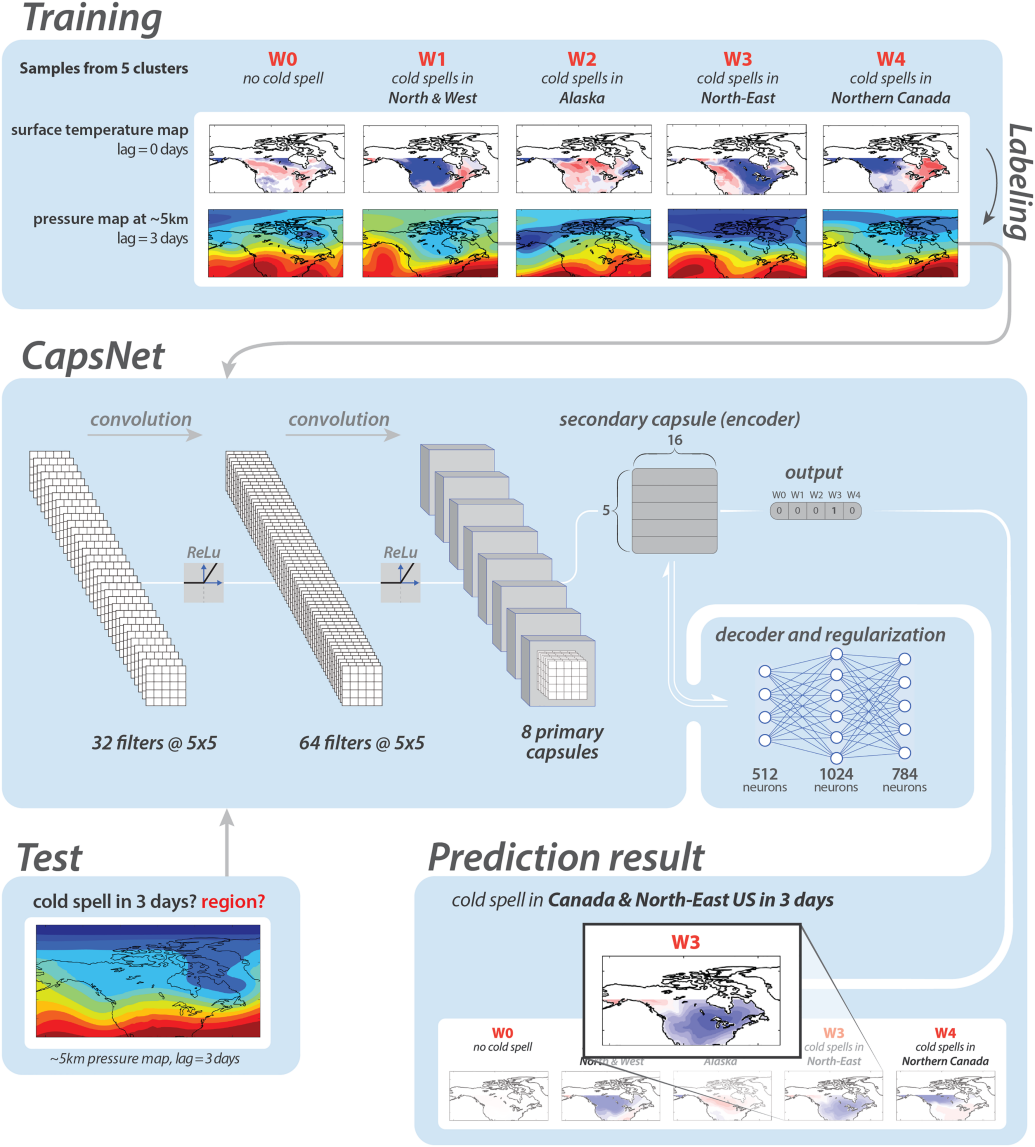
Schematic of the data‐driven framework for prediction of cold spells based on Z500 patterns of 3 days earlier. Using the impact‐based autolabeling strategy, Z500 patterns are labeled W0, W1, W2, W3, or W4, depending on the cluster index of T2m 3 days ahead. The panels at the top show examples of T2m patterns at the onset and the corresponding Z500 patterns (from 3 days earlier) for each cluster. Only the Z500 patterns and their labels are inputted into the CapsNet during training. Once trained, the CapsNet can predict, from a given Z500 pattern, the T2m cluster index of 3 days later, thus predicting the occurrence and geographical region of cold spells. For the shown test example, a cold spell in W3 in 3 days is predicted. Note that for winters, Z500 patterns over a larger domain that extends across the Pacific Ocean to 145°E are inputted into the CapsNets, but a smaller domain is shown in this figure for better illustration (see Section 3.2). Separate CapsNets are trained for each season and each prediction lead time.

We use two state‐of‐the‐art deep learning techniques for pattern recognition: convolutional neural network (ConvNet) (Goodfellow et al., 2016; LeCun et al., 2015) and a more advanced method, capsule neural network (CapsNet) (Sabour et al., 2017). The key advantage of both methods over traditional image‐processing techniques is that the filters used for feature extraction are learned for each data set through an algorithm called backpropagation (Goodfellow et al., 2016), rather than being hand‐engineered and specified beforehand. ConvNet is the groundbreaking method that has transformed image processing since 2011, but because of a property called equivariance that is discussed later, CapsNet is expected to work even better for our spatiotemporal climate data. Figure 2 shows the architecture of the CapsNet schematically. Details of the ConvNet and CapsNet architectures are presented in Appendices A and B, respectively.

### 3.2 Impact‐Based Autolabeling of Daily Z500 Patterns

Both ConvNet and CapsNet are supervised methods, meaning that they have to be first trained on labeled patterns. However, given the incomplete understanding of extreme‐causing weather patterns and their complexities, expert‐labeled data are not useful for our objective. For example, indices designed to find blocking patterns in the Z500 field based on their presumed properties are known to perform poorly, for example, in identifying extreme‐causing patterns even on the same day as the heat or cold extreme events (Chan et al., 2019). Expert‐labeling becomes even less effective for the purpose of prediction, which requires accounting for the nonlinear spatiotemporal evolution of the atmospheric circulation over several days or longer.

**Figure 3:**
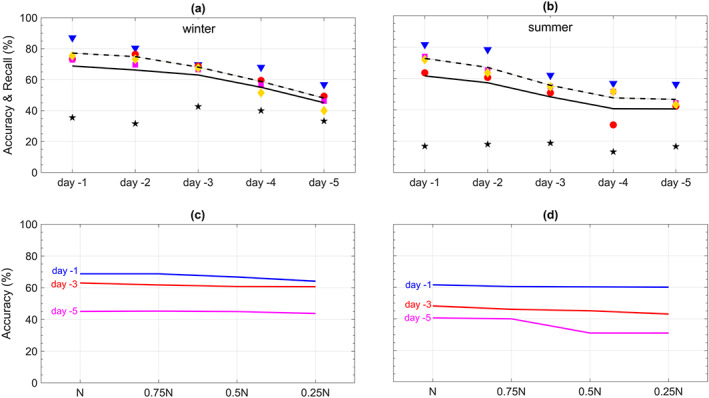
Performance of CapsNets in predicting heat waves and cold spells using Z500 patterns at various lead times. (a and b) The symbols show the accuracy at different lead times for each cluster: star (0), triangle (1), square (2), diamond (3), and circle (4). The solid (dashed) lines show the total accuracy (recall). (c and d) Total accuracy at prediction lead times 1, 3, and 5 days versus the size of the training set ( N=750; fractions are rounded to the nearest integer if needed). Results in (a) and (b) are obtained with the largest training set. The symbols show the accuracy averaged over three randomly drawn pairs of training/testing sets. The lines and their shading depict the mean and ±1SD of accuracy or recall computed for the three pairs; the shadings are narrow, demonstrating the small SD and robustness of the results.

**Figure 4:**
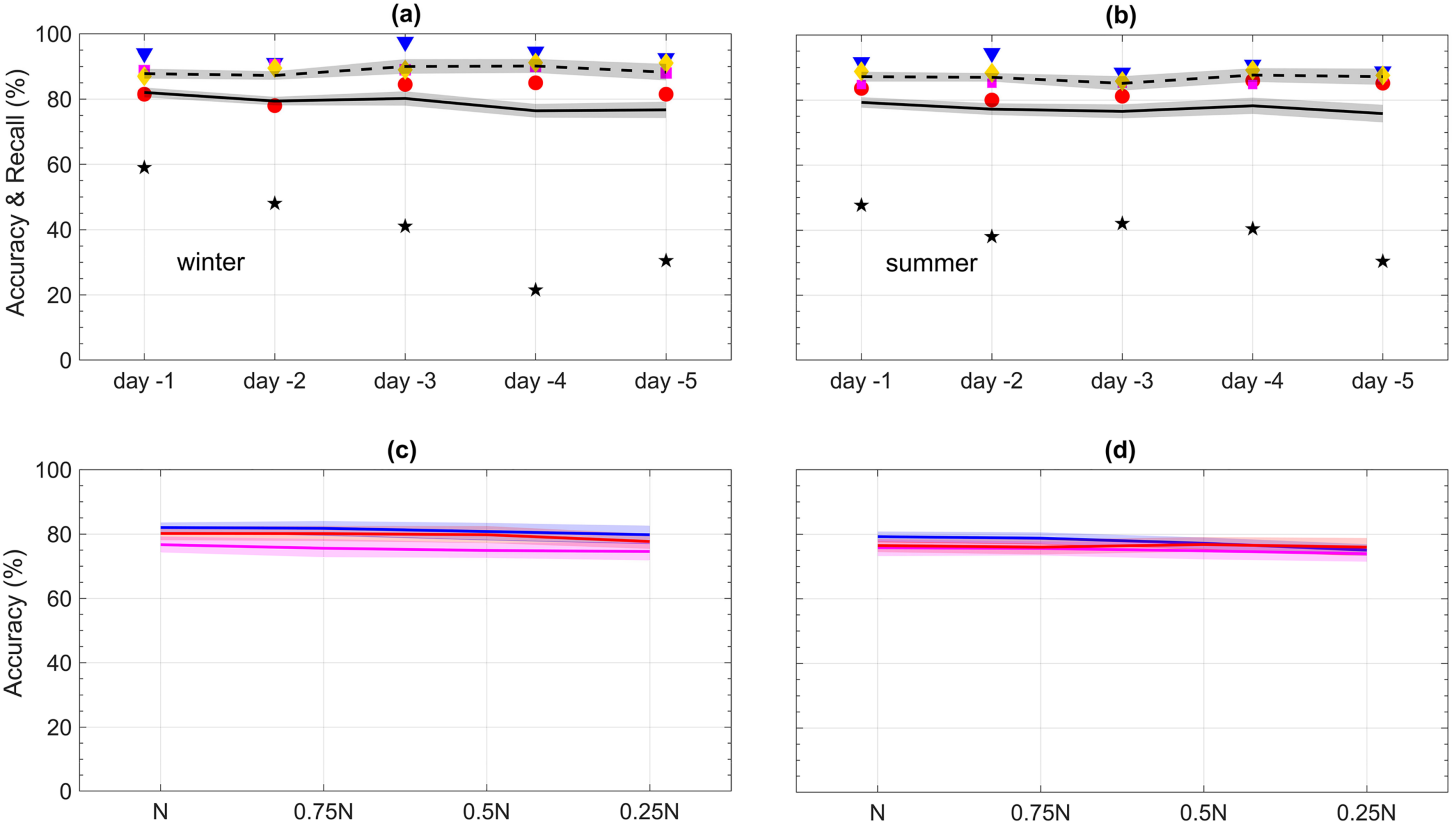
Same as Figure 3 but for the performance of CapsNets in predicting heat waves and cold spells using both T2m and Z500 patterns. The shadings show ±1SD; the SD values are higher in the multivariate approach. In (c) and (d), the change of accuracies with the size of the training set is small for lead times of 1, 3, and 5 days (labels not shown).

**Figure 5:**
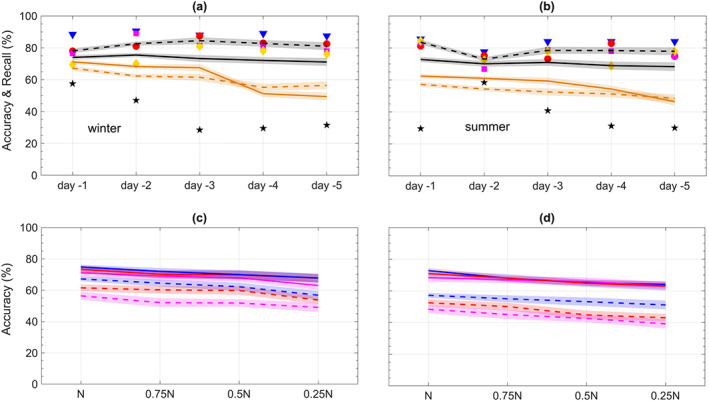
Same as Figure 3 but for the performance of ConvNets and logistic regression in predicting heat waves and cold spells using Z500 patterns at various lead times. In (a) and (b), the symbols and black lines correspond to ConvNets, while the green lines correspond to logistic regression. In (c) and (d), the solid (dashed) lines correspond to ConvNets (logistic regression).

**Figure 6:**
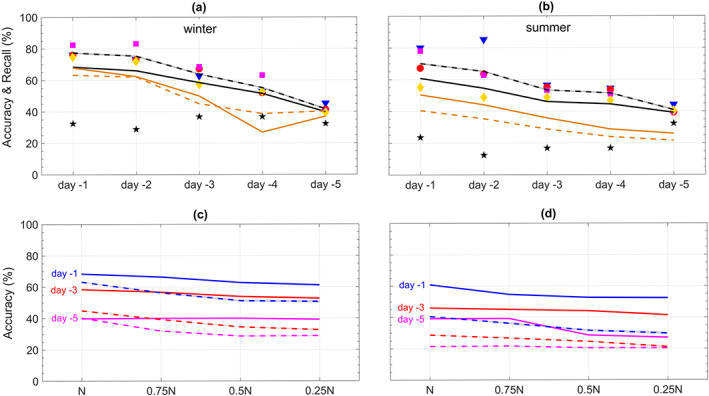
Same as Figure 4 but for the performance of ConvNets and logistic regression in predicting heat waves and cold spells using both T2m and Z500 patterns at various lead times. In (a) and (b), the symbols and black lines correspond to ConvNets, while the green lines correspond to logistic regression. In (c) and (d), the solid (dashed) lines correspond to ConvNets (logistic regression).

To overcome this challenge, here we devise an impact‐based autolabeling strategy: Knowing the surface temperature over North America on a given day, the Z500 pattern of several days earlier is labeled as 0 (no extreme onset) or 1, 2, 3, or 4 (the cluster indices of T2m extremes). For example, for predicting heat waves in summers with a lead time of 3 days, the Z500 patterns 3 days before the onsets are labeled S0–S4 based on the cluster index of the T2m at the onsets. Rows 2 and 4 of Figure 1 show the average of Z500 patterns with the same labels for 3‐day prediction. For the Z500 patterns, we use two domains with the same latitudinal (30–90°N) but different longitudinal extents: 195–315°E (small domain, shown in Figures 1 and 2) and 145–340°E (large domain). For winters, at prediction lead time beyond 2 days, we have found higher accuracy and recall with the large domain, which is likely due to the dominance of zonal advection and Rossby wave propagation in the evolution of weather patterns. For summers, we have found higher accuracy with the small domain for all prediction lead times. The results in Figures 3, 4, 5, 6 are with large (small) domain for winters (summers).

We highlight that in conventional deep learning applications, labeling and training/testing are all conducted on the same feature map; however, in the impact‐based labeling strategy introduced here, we label based on differences in one feature map (T2m) but train/test on another feature map (Z500), in order to predict the original feature map (T2m). While more challenging, the impact‐based autolabeling strategy circumvents the need for a full understanding of the complex and nonlinear relationship between the predictor (Z500) and the impact of interest (T2m).

### 3.3 Training and Testing Data Sets

For each season and prediction lead time, we build a data set of M labeled Z500 patterns per cluster. While the number of onsets varies among the clusters and there are many more nonextreme events than extreme events, to avoid *class imbalance*, we choose M near the smallest number of onsets among the four clusters of extreme events. For both summer and winter, we use M=1,000 for prediction lead times of 1 and 2 days and M=900 for longer lead times. For each pair of training and testing sets, we randomly choose N=3M/4 samples per cluster for the training set and the remaining M/4 samples for the testing set. Note that the total number of samples in the training and testing sets ( ∼4,500–5,000) is much lower than the total number of summer or winter days (316,480 and 306,000, respectively) in the LENS data set, because we are focusing on the rare events (i.e., onsets of extremes in the 99th or 1st percentile). We also report the accuracies with smaller training sets of sizes 3N/4, N/2, and N/4 samples per cluster in Figures 3, 4, 5, 6 (the number of samples in the testing set is kept as M/4 regardless of the size of the training set).

### 3.4 Data‐Driven Extreme Weather Prediction Framework

The schematic of the entire data‐driven prediction framework is shown in Figure 2. Separate CapsNets (or ConvNets) are trained and used for different seasons and prediction lead times (referred to as Cases hereafter). For example, Figure 2 shows the framework with CapsNet for prediction of cold spells in winter with a 3‐day lead time. During the training phase, the same number of Z500 patterns ( N per cluster) and their cluster indices from the training set are inputted into the CapsNet. The trained CapsNet can then predict the cluster index of a never‐seen‐before Z500 pattern inputted from the testing set. If the output of CapsNet is index W0, then no cold spell anywhere over North America (between 30°N and 60°N) is forecasted in 3 days, while other outputs indicate prediction of a cold spell in 3 days over Northwest United States‐Canada (W1), Alaska (W2), Northeast United States‐Canada (W3), or Northern Canada (W4).

The above framework for predicting the extreme‐causing weather patterns (just based on information from Z500) is used for the results of Figures 3 and 5. To further demonstrate the potentials of this data‐driven framework in predicting the extreme events in a multivariate approach, we have also shown results in Figures 4 and 6 with an extended framework, in which the inputs consist of the patterns of both Z500 and anomalous T2m stacked together in two different channels. Due to the difference in their mean and variability, standardization is performed on each channel separately.

### 3.5 Training and Testing Procedure and Accuracy/Recall Calculations

For each case, the M labeled samples are randomly divided into a training set and testing set (with ratio of 3:1, as discussed above) four times to create four pairs of training/testing sets. One pair is used as a validation set to explore the CapsNet and ConvNets' hyperparameters such as kernel size, regularization constant, and dropout probability. For each Case, once a suitable set of hyperparameters is found, the CapsNet (or ConvNet) is trained and tested just once on each of the remaining three pairs of the training/testing sets (to emphasize, the hyperparameters are not changed for these three pairs, but the weights of the filters are learned independently each time). The accuracies and recalls reported in Figures 3, 4, 5, 6 and in the text are computed as the average of results with these three pairs of data sets. We have adopted this approach to examine the robustness of the results despite the relatively small size of the labeled data set.

We report the prediction skills in terms of the total accuracy of the testing set, computed as the number of test samples from all five clusters whose cluster index is correctly predicted divided by the total number of test samples, and recall, computed as the number of test samples from the four clusters with extreme events (1–4) whose cluster index is correctly predicted divided by the total number of test samples in Clusters 1–4. We computed the recall because for extreme weather prediction, missed events are much more undesirable than false alarms. Together, accuracy and recall fully quantify the skills of the framework for a multiclass prediction. Note that the accuracy for individual clusters, computed as the number of correctly predicted test samples from that cluster divided by the total number of test samples from that cluster, is the receiver operating characteristic score, a common forecast skill metric (Fawcett, 2006; McKinnon et al., 2016).

## 4 Results

Figure 3 shows the performance of CapsNet for predicting cold spells and heat waves using the Z500 patterns from 1–5 days earlier. The accuracies for lead times of 1 to 5 days are between 68.8% ± 0.3% and 45.1% ± 0.1% in winter, and 61.6% ± 0.0% and 40.6% ± 0.1% in summer, against a 20% random chance in a five‐class prediction. The recalls are consistently higher, between 77.2% ± 0.3% and 48.1% ± 0.1% in winter and 72.8% ± 0.1% and 46.6% ± 0.1% in summer. Examining the prediction accuracy for individual clusters shows that the inaccuracies largely result from false alarms due to nonextreme events (Cluster 0) incorrectly predicted as an extreme event somewhere in North America (Clusters 1–4). False alarms can be reduced by adding more constraints on Z500 during labeling, for example, requiring daily Z500 anomalies to exceed 1.5 standard deviation (SD); however, we choose to avoid subjective criteria and only use the impact (i.e., T2m extreme) for labeling. Furthermore, we focus on minimally preprocessed inputs, for example, we do not detrend Z500 patterns and instead use the full Z500 patterns (see Section 3.2), which are nonstationary due to low‐frequency coupled atmosphere‐ocean modes of climate variability and changes in the radiative forcing from 1920–2005.

The results in Figures 3a and 3b are obtained with a training set containing N=750 samples from each of the five clusters. Figures 3c and 3d show that as the size of the training set is reduced, the accuracies for winter barely decline. Even when the number of training samples per cluster is reduced almost by a factor of 4 to 187 or 168 (depending on the lag), the largest decrease in accuracy is 4.7% (for Day −4). In summer, the effect of the size of the training set is more pronounced especially at longer lead times, for example, the accuracy for 5‐day prediction declines by 9.5% when the training set is reduced by a factor of 4. Overall, the weak dependence of the accuracy on the size of the training set is encouraging for practical purposes (see Section 5), but it also suggests that likely, higher accuracies could not be achieved even if we had more training samples (see below).

The prediction skills in summers are lower than those in winters. Figure 1 shows that the Z500 patterns corresponding to different clusters are much more similar in summers than in winters, suggesting that it should be harder to identify the correct cluster of a pattern in summer. Still, that CapsNet can differentiate between patterns that have such similar averages (i.e., cluster centers) with the accuracy (recall) of 48.2% ± 0.1% (55.6% ± 0.1%) shows the effectiveness of the framework. Furthermore, dynamics of heat waves are more complex than cold spells and the midtropospheric circulation patterns (the only predictor here) are not the only driver: Cold spells are mostly due to equatorward advection of cold air from higher latitudes while the heat waves are caused by a combination of horizontal advection and adiabatic and clear‐sky radiative warmings (Dole et al., 2011; Pfahl & Wernli, 2012; Schneider et al., 2015). Moreover, land‐atmosphere feedbacks play a role in the dynamics of heat waves (Miralles et al., 2014).

The results of Figure 3 show the power of our data‐driven framework for predicting the surface temperature extreme events using a single variable (Z500) that represents midtropospheric circulation, that is, predicting extreme‐causing weather patterns. The above discussions on the weak dependence of accuracy on the size of the training set and the dynamics of the extreme temperature events suggest that including more variables as the predictor and pursuing a multivariate framework would lead to better prediction skills for the extreme temperature events, particularly at longer lead times. It should be highlighted that even for winters, where meridional advection dominates, including information from other altitudes of troposphere and stratosphere (e.g., to account for polar vortex variability) are expected to improve the prediction skills (see Section 5).

To demonstrate the promises of such multivariate data‐driven frameworks, we repeat the analysis of Figure 3 but by inputting the patterns of Z500 and anomalous T2m together into CapsNet in the training and testing phases. Figure 4 shows that the accuracies (recalls) for lead times of 1 to 5 days rise to between 82.0% ± 1.5% (87.8% ± 1.4%) and 76.7% ± 2.5% (88.2% ± 2.3%) in winter and 79.3% ± 1.6% (87.2% ± 1.7%) and 75.8% ± 2.7% (87.2% ± 2.6%) in summer, significantly improving the prediction skills, particularly in the longer lead times. With T2m included, the false alarms decline in most cases, and the accuracies/recalls hardly change with lead time or size of the training set. It should be highlighted that the high prediction skills with Z500+T2m are not simply due to the temporal memory as a result of including T2m of earlier days. With N=750, training and testing the CapsNets on T2m alone result in accuracies that are consistently lower, between 0.6% and 5.2% (1.5–4.5%), than the accuracies with Z500+T2m in winter (summer), showing that information about the atmospheric circulation adds to the prediction skills.

The accurate and robust 1‐ to 5‐day predictions in Figure 4 suggest that the multivariate framework using Z500+T2m, or even more variables (see Section 5), might have high prediction skills for lead times beyond 5 days. However, such longer predictions will require using Z500 patterns (and some of the other variables) at the planetary scales, which, for the best performance of the framework, needs CapsNet (and ConvNet) architectures capable of accounting for the Earth's spherical geometry, for example, the zonal periodicity and decrease of area with latitude. Extending the framework to planetary scales and longer prediction lead times is left for future work, which will benefit from recent advances in developing spherical ConvNets (Cohen et al., 2018; Jiang et al., 2019).

We also conduct the analyses in Figures 3 and 4 with CapsNet replaced with two simpler methods: (i) ConvNet, which is a deep learning method of growing interest in climate science (and other disciplines) and was used, for example, by Liu et al. (2016) to identify tropical cyclones and atmospheric rivers and by Ham et al. (2019) for multiyear El Niño–Southern Oscillation prediction, and (ii) logistic regression (Goodfellow et al., 2016), which is a widely used machine learning method that has been employed in some past weather forecasting efforts (Applequist et al., 2002; Chattopadhyay et al., 2020; Herman & Schumacher, 2018; Whan & Schmeits, 2018). Figures 5 and 6 show that the CapsNets consistently outperform the ConvNets (except for one Case: 4‐day lead time in summer). For predictions with Z500 (Z500+T2m), the accuracies of CapsNets are, on average, higher than ConvNets by 2.8% (7.7%) in winters and 0.7% (7.1%) in summers. More importantly, as the size of the training set is reduced, the accuracy of ConvNets degrades more than that of CapsNet, particularly in the multivariate approach with Z500+T2m (Figures 5c, 5d, 6c, and 6d). Due to their different architectures (see Appendices A and B), CapsNets extract more features and information from each pattern compared to ConvNets and are thus expected to work well even with relatively small training sets. Moreover, CapsNets account for the relative position and orientation of features (a property called equivariance) (Sabour et al., 2017). Relative positions of features in spatiotemporal climate data are important; for example, high‐pressure systems on the poleward side of low‐pressure systems might stall and cause weather extremes, while low‐pressure systems on the poleward side of high‐pressure system often move eastward without causing extreme events.

The accuracy of logistic regression is consistently lower than that of ConvNets (and thus CapsNet); see Figures 5 and 6. For predictions with Z500, the accuracies of CapsNets are, on average, higher than those of logistic regression by 11.4% (19.6%) in winters (summers). These results show the advantage of more advanced deep learning techniques over simpler ones such as ConvNet and logistic regression and suggest that future studies in climate and environmental sciences might benefit from using CapsNets (and might benefit even more from deep learning techniques designed specifically for multiscale, spatiotemporal, chaotic data).

Note that we did not compare the performance of our framework with persistence or climatology, which are two common baseline methods (Murphy, 1992), because they could not be formulated to predict T2m extremes based on inputs of Z500 patterns, and that by definition, there is no T2m extreme within 5 days of the onsets (see Appendix C).

## 5 Discussion

The results of Figure 3 show the skills of the data‐driven framework in predicting high‐impact events (e.g., T2m extremes) only through limited information about the events's driver (or one of the key drivers), that is, Z500 patterns in this case, and without any information about the impact itself. This skillful prediction of extreme‐causing weather patterns provides a proof of concept for the framework (Figure 2). We emphasize that the key components of this data‐driven framework are the novel impact‐based autolabeling technique and the power of CapsNets in pattern recognition, which together enable the framework to decipher the relationship between the T2m and Z500 patterns and the temporal evolution of Z500 patterns, despite challenges such as sensitivity of nonlinear systems to small perturbations in initial conditions (Lorenz, 1969) and the rarity of perfect analogs in climate data (Van den Dool, 1994, 2007).

Based on the results of Figure 4, the multimodal framework (in which both Z500 and T2m are used together), once equipped with spherical CapsNets for planetary‐scale inputs, may offer a promising data‐driven approach to prediction. Higher accuracies and longer prediction lead times (at the weekly to seasonal time scales, which are of the most utility and interest) might be achievable by including variables such as geopotential heights at more tropospheric levels, soil moisture, and outgoing longwave radiation, as well as information from the slow‐varying boundaries of the troposphere such as tropical and extratropical sea surface temperature (e.g., from Pacific Ocean), tropical atmosphere (e.g., Madden‐Julien Oscillation), sea ice, and stratosphere, which are all known to enhance predictive skills for the midlatitude extreme events (Baldwin & Dunkerton, 2001; McKinnon et al., 2016; Mundhenk et al., 2018).

The data‐driven extreme event prediction framework introduced here can be useful in (at least) two ways: (1) to provide early warnings of extreme events and guide the public and NWP efforts and (2) to identify the precursors of extreme events using ideas from interpretable machine learning (Zeiler & Fergus, 2014). Regarding the former, one of the most appealing and powerful aspects of a data‐driven framework is the possibility of training on observational data. Here, the main challenges in using observed climate data for training are that such records are short, and the data are nonstationary in time. Reanalysis products are available for as early as 1850, although the data from before 1979 are derived from limited direct observations. The LENS data used in this study have complexity and nonstationarity similar to that of the reanalysis data; however, the 40‐member ensemble simulations provide, for example, ∼300,000 days of data in winters, which is much larger than what can be obtained from reanalysis data sets. Given our focus on the onsets of extreme events in the 1st or 99th percentile, from the LENS data, we only used as high as 750 and as low as 168 samples per cluster for training. Figures 3 and 4 show that even with the smallest training set, skillful multiclass predictions are obtained. Furthermore, transfer learning can be used to first train the CapsNet (or ConvNet) on a large set of modeled data and then on a small set of reanalysis, as, for example, recently done by Ham et al. (2019) for El Niño–Southern Oscillation forecasts. The above discussion suggests that it might be possible to use data derived from observations (and not just NWP or climate models) for training of the data‐driven framework. We highlight again that the purpose of this paper is to provide a proof of concept for the framework. Evaluating the performance of the framework trained on reanalysis data and comparing the forecast skills with those of the NWP models are admittedly the essential and important next steps, and are currently underway.

Data from LENS, high‐resolution NWP model simulations can also be used for training. The very high resolution NWP models, which need prohibitive computing time/resources to run continuously, simulate the atmospheric circulation, and in particular extreme‐causing patterns, with higher fidelity compared to the simulations with lower resolutions (Alley et al., 2019; Jung et al., 2012). The advantage of using the data‐driven framework, trained on high‐resolution NWP models, is that it can yield extremely fast and inexpensive regional predictions, which can provide early warnings and guide the deployment of computing and sensing resources for LENS, high‐resolution NWP of a region predicted to experience an extreme event in several days (or longer).

In this study we conducted five‐class predictions based on extreme events over North America clustered (using K‐means algorithm) into four geographical regions. However, other clustering algorithms, number of clusters, etc. could be used, or alternatively, separate data‐driven frameworks can be developed for binary (yes/no) extreme predictions in each region of interest, for example, around Greater Houston, Bay Area, Greater Boston. Understanding which of the approaches discussed above (differing in training data and framework design) lead to the best data‐driven prediction skills and better handle practical limitations requires extensive research and should be addressed in future studies.

Precursors of extreme‐causing weather patterns such as blocking events are not well understood (Hassanzadeh & Kuang, 2015; Nakamura & Huang, 2018; Woollings et al., 2018) and identifying them can lead to a better dynamical understanding of extreme events and potentially improving weather and climate models. Given that CapsNets show skills in predicting the extreme‐causing weather patterns, it is of interest to understand how the neural network has *learned* what key features to look for. However, understanding how deep neural networks work is known to be challenging and an active area of research. In future work, the feature maps and filters should be examined to seek an understanding of how CapsNets differentiate between patterns that in a few days lead to different T2m clusters. Recent papers by Ham et al. (2019) and McGovern et al. (2019) present promising results and discuss interesting ideas on interpretation of ConvNets and other machine learning methods applied to climate data.

Finally, our data‐driven framework can be readily generalized for prediction of other high‐impact climatic and environmental phenomena, for example, extreme precipitation or severe air pollution events, just to name a few. The impact‐based autolabeling strategy circumvents the need for a full understanding of the relationship between the impact of interest and its driver(s). Needless to say, domain expertise is still critical in designing the autolabeling strategy, for example, in choosing the relevant variables and spatiotemporal scales.

## Appendix A Convolutional Neural Network (ConvNet)

### A.1

ConvNet has transformed image processing and pattern recognition in recent years, particularly after its performance in analyzing the ImageNet data set in 2011 (Krizhevsky et al., 2012), a turning point in the history of deep learning (Goodfellow et al., 2016; LeCun et al., 2015). The main components of a ConvN et algorithm are as follows: convolutional layers, in which a specified number of filters with specified sizes extract the key features in the data and produce feature maps; Rectified Linear Unit (ReLU) layers, in which the activation function f(x)=max(0,x) is applied to the feature maps to introduce nonlinearity; pooling layers, which reduce the dimensions of the feature maps by taking the maximum or average of adjacent data points, are applied to increase computing efficiency, control overfitting, and induce translational and scale invariance; and fully connected layers (Goodfellow et al., 2016; LeCun et al., 2015). During the training phase, patterns and their labels are inputted into ConvNet and the filters (i.e., their weights) are learned using backpropagation.

For the small (large) domain, the Z500 patterns are on a 97×66 (157×66) longitude‐latitude grid. In our earlier work on applying ConvNet to the LENS data set (Chattopadhyay et al., 2020), we found the training/testing on the full‐size patterns challenging. This is because the high‐frequency, small‐scale variability, for example, associated with baroclinic waves, is highly chaotic and the ConvNets' attempts in learning these features are futile and lead to substantial overfitting and inaccuracies. We further found that this problem can be resolved by first downsampling the data to 28×28 (using bicubic interpolation), which only retains the large‐scale features. See Chattopadhyay et al. (2020) for further discussions. The downsampled patterns are then standardized by removing the mean and normalizing by the standard deviations computed over the training and testing sets combined.

Our ConvNet architecture is similar to the one used in Chattopadhyay et al. (2020). There are four convolutional layers, which have 8, 16, 32, and 64 filters, respectively, and each filter has a kernel size of 5×5. Zero padding is used to maintain the size of patterns before and after applying the filters. Each convolutional layer is followed with a ReLU activation function. For the last two convolutional layers, the ReLU layers are followed by max‐pooling layers that have a kernel size of 2×2 and stride of 1. The output feature is fed into a fully connected neural network with 200 neurons. The cross‐entropy cost function is accompanied by a $L_{2}$ regularization term, and to prevent overfitting, dropout regularization has been used in the fully connected layer. An adaptive learning rate is implemented through the ADAM optimizer (Kingma & Ba, 2014). The final output is the probability of the input pattern belonging to each cluster. A softmax layer assigns the pattern to the cluster index with the highest probability. Chattopadhyay et al. (2020) provide further details on the hyperparameter choices, optimizing the ConvNet, and the effect of the number of convolutional layers (e.g., using only two layers reduces the performance while using eight leads to overfitting).

## Appendix B Capsule Neural Network (CapsNet)

### B.1

Despite its groundbreaking performance and ever‐growing popularity, ConvNets face difficulty in dealing with some types of problems where the spatial relationship of key features become critical; for example, a cubist painting of a face that has the position of one eye and the mouth switched has the right features of a face but not at the right relative positions. The ConvNet's challenge in dealing with these problems is due to the pooling layers, which as mentioned earlier, take the maximum or average of the adjacent data points to introduce translational and scale invariances in the deeper layers, so that ConvNet can recognize the same object at a somewhat different location or with a different size. To address this issue, Sabour et al. (2017) have introduced CapsNet, which does not have pooling layers but have capsules, which are nested sets of neural layers. Unlike a neuron whose output is a real‐valued scalar (e.g., likelihood of a certain feature), the output of a capsule is a vector consisting of more information (e.g., position, relative distances, poses, and orientation). Rather than invariance, CapsNet seeks equivariance, meaning that the relative positions of features are encoded in the feature maps.

One might expect CapsNet to be more suitable than ConvNet for our data‐driven extreme weather prediction framework (and other climate and environmental problems), because of the importance of the relative position of features. For example, a high‐pressure system on the poleward side of a low‐pressure system, or a high‐pressure system on the poleward side of two weaker low‐pressure systems in an Omega‐shape configuration, is the signature structures of persistent and extreme‐causing blocking events (Woollings et al., 2018); however, the opposite configurations, for example, a low‐pressure system on the poleward side of a high‐pressure system, often progress eastward and do not cause extreme events.

The architecture of our CapsNet is similar to that of Sabour et al. (2017) and is shown in Figure 1. There are two convolutional layers with 32 and 64 filters with kernel sizes 5×5, followed by ReLU layers. These are followed by a primary capsule layer (with eight capsules, each with eight convolution layers) that encodes the information of the features in the form of high‐dimensional tensors. Via the routing‐by‐agreement algorithm, the information is sent from the primary capsule layer to a secondary capsule layer, where the probability of each cluster is predicted. At each capsule, nonlinearity is introduced by a squash function (Sabour et al., 2017) that enforces the output to have a magnitude less than one while preserving its orientation. There is also a decoding layer, a novel regularization algorithm, in which the pattern is reconstructed using three fully connected layers to its original size ( 28×28) to penalize the loss function to enforce the search for feature maps that can closely reconstruct the original pattern.

## Appendix C Other Skill Metrics: Persistence and Climatology

### C.1

Two commonly used baseline methods for forecasting skills are climatology (wherein a variable on a particular date is forecasted as the long‐term average of that variable on that particular calendar date from previous years) and persistence (where we assume that the variable remains the same over the period the forecast is performed) (Murphy, 1992). We have not compared the performance of our framework with these baseline methods because our objective could not be formulated in terms of these methods.

To predict T2m based on Z500 alone, climatology and persistence are inapplicable because they can predict Z500 patterns (or cluster) based on earlier Z500 patterns but cannot predict T2m pattern (or cluster) based on the input of Z500 patterns, which is our objective here. To predict T2m based on Z500+T2m, persistence could not be used because by definition, there is no T2m extreme within 5 days of any temperature extreme onset. Climatology cannot be used because we have defined extremes as the 1st or 99th percentile of climatological temperature, far from the average.
